# Pannexin-1 in Human Lymphatic Endothelial Cells Regulates Lymphangiogenesis

**DOI:** 10.3390/ijms19061558

**Published:** 2018-05-24

**Authors:** Jonathan Boucher, Claire Simonneau, Golthlay Denet, Jonathan Clarhaut, Annie-Claire Balandre, Marc Mesnil, Laurent Cronier, Arnaud Monvoisin

**Affiliations:** 1 CNRS ERL 7003, Laboratoire “Signalisation & Transports Ioniques Membranaires”, University of Poitiers, 86073 Poitiers, France; jonathan.boucher@univ-poitiers.fr (J.B.); claire.simonneau86@gmail.com (C.S.); golthlay.denet@etu.univ-poitiers.fr (G.D.); annie-claire.balandre@univ-poitiers.fr (A.-C.B.); marc.mesnil@univ-poitiers.fr (M.M.); Laurent.Cronier@univ-poitiers.fr (L.C.); 2 CNRS UMR 7285, Institut de Chimie des Milieux et des Matériaux de Poitiers (IC2MP), University of Poitiers, 86073 Poitiers, France; jonathan.clarhaut@chu-poitiers.fr; 3 CHU de Poitiers, 86021 Poitiers, France

**Keywords:** lymphatic endothelial cells, pannexins, Panx1, lymphangiogenesis, cell invasion, Vascular Endothelial Growth Factor-C (VEGF-C)

## Abstract

The molecular mechanisms governing the formation of lymphatic vasculature are not yet well understood. Pannexins are transmembrane proteins that form channels which allow for diffusion of ions and small molecules (<1 kDa) between the extracellular space and the cytosol. The expression and function of pannexins in blood vessels have been studied in the last few decades. Meanwhile, no studies have been conducted to evaluate the role of pannexins during human lymphatic vessel formation. Here we show, using primary human dermal lymphatic endothelial cells (HDLECs), pharmacological tools (probenecid, Brilliant Blue FCF, mimetic peptides [^10^Panx]) and siRNA-mediated knockdown that Pannexin-1 is necessary for capillary tube formation on Matrigel and for VEGF-C-induced invasion. These results newly identify Pannexin-1 as a protein highly expressed in HDLECs and its requirement during in vitro lymphangiogenesis.

## 1. Introduction

Pannexin-1 (PANX1) is one of the three members of the Pannexin family with PANX2 and PANX3 discovered through homology to the invertebrate gap-junction forming proteins, innexins [1,2]. Pannexins (PANXs) and Connexins (CXs) share similar protein structure while they lack amino acid sequence homology [3]. By hexameric oligomerization PANX1 forms unopposed large-pore channels [4,5] which allow the release of molecules up to 1 kDa into the extracellular space such as ions, adenosine triphosphate (ATP) and other nucleotides [6,7]. PANX1 is ubiquitously expressed in several organs and tissues [2,8,9,10,11,12] and is the best characterized isoform of the PANX family. For a long time, PANX2 expression has been restricted to the central nervous system [13], but it is now well described that PANX2 is also ubiquitously distributed throughout the body [14]. Similarly, PANX3 has been mainly described in cartilage, bone and skin [2,11,15,16,17,18,19,20,21] but accumulating evidences show PANX3 expression in other tissues such as skeletal muscle, heart, cochlea, and arteries [2,15,18,22,23].

In line with the large PANX1 tissue distribution, this ATP release channel is directly or indirectly involved in numerous physiological functions or pathologies such as inflammatory diseases [24] or cancer [25,26]. However, little is currently known regarding the role of PANX1 in the vasculature. PANX1 has been found to be expressed in vivo within the vascular wall in arteries, arterioles, capillaries, veinules and smooth muscle cells (SMCs) but not in veins [12,27] and in vitro in isolated blood endothelial cells (ECs) [28,29,30]. Panx1-deficient mice showed significantly impaired endothelial function [31]. ATP release via PANX1 channels by either ECs or SMCs has been involved in the regulation of vascular tone, inflammation, and cerebral ischemic stroke [30,32,33]. While many data are available in blood vascular system, studies regarding roles of Panxs in regulating lymphatic vascular development are currently missing [34]. The main role of the lymphatic system is to transport in a unidirectional way extravasated fluids and macromolecules from tissues, through lymph nodes, back to the blood circulation to maintain homeostasis [35]. Lymphangiogenesis, the formation of new lymphatic vessels from preexisting ones [36,37], is associated with several diseases such as chronic inflammation, graft rejection and metastatic dissemination [38]. Since the identification of the Vascular Endothelial Growth Factor-C (VEGF-C) as the major lymphangiogenic factor [39], several other key genes and proteins involved in lymphatic development have been identified. Among them, it has recently been shown that at least three CX isoforms (CX37, CX43 and CX47) are expressed in developing and mature lymphatic vessels [40,41,42]. These studies showed that these CXs are necessary for the proper lymphatic valve development in collecting vessels and contribute to morphogenesis of the jugular lymph sac and thoracic duct. Moreover, CX mutations or deficiency in mouse and humans have been found to lead to lymphedema [40,43,44]. Regarding PANXs, no in vitro nor in vivo data were available onto their expression and/or function in human lymphatic vasculature and only one recent study has shown the expression of Panx1 in mouse LECs by qPCR [45]. This study aims to investigate the expression of PANXs in human lymphatic endothelial cells and more particularly the role of PANX1 during lymphangiogenesis.

## 2. Results

### 2.1. Human Lymphatic Endothelial Cells Express Pannexins

**Figure 1:**
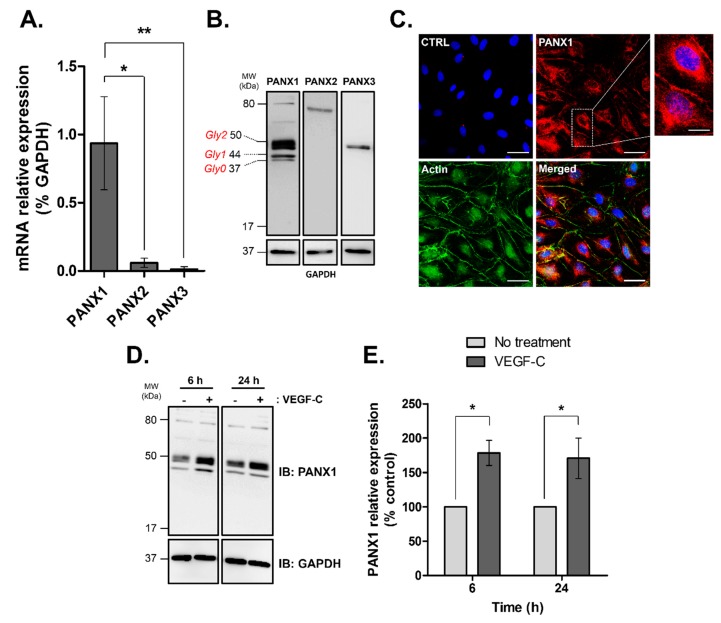
Pannexin isoforms expression in human dermal lymphatic endothelial cells (HDLECs). (**A**) PANXs mRNA expression in isolated HDLECs quantified by RT-PCR and normalized by GAPDH. The data represent mean ± SD from three independent experiments; (**B**) Western blot analysis of total protein extracts (20 μg/lane) from four independent HDLEC cultures demonstrating PANXs expression in HDLECs. Unglycosylated (Gly0) and glycosylated isoforms (Gly1 and Gly2) of PANX1 are indicated; (**C**) PANX1 immunofluorescence in HDLECs (red), F-actin was FITC-phalloidin stained (green) and nuclei were DAPI-stained (blue). CTRL: control immunofluorescence after omission of the primary antibody, Scale bar: 50 μm; Enlarged image marked by the white box shows higher magnification of PANX1 staining, scale bar 7 µm; (**D**) Representative Western blot analysis and (**E**) densitometric quantification of PANX1 expression normalized to GAPDH following 100 ng/mL VEGF-C treatment for the indicated times in HDLECs. Values are expressed as mean ± SD from three independent experiments. * *p* < 0.05 and ** *p* < 0.01.

PANX1, -2 and -3 mRNA expression was examined by quantitative RT-PCR in human lymphatic endothelial cells. As shown in Figure 1A, the expression of *PANX1* was highest among the 3 PANX gene family while *PANX2* and *PANX3* were barely expressed.

Interestingly, Western blot analysis demonstrated that all three PANX isoforms were expressed in the HDLECs (Figure 1B). All PANXs were detected at the expected molecular weight. As previously described, a specific banding pattern of three bands was revealed for PANX1 indicating three different glycosylation states: Gly0, non-glycosylated core protein; Gly1, high-mannose species and Gly2, complex glycosylated species [4,46].

Confocal imaging of HDLECs showed a clear localization of PANX1 to the plasma membrane and in the perinuclear compartment (Figure 1C). Importantly, we found that VEGF-C, the main regulator of lymphangiogenesis, increased PANX1 expression in HDLECs after 6 and 24 h treatment by 78 ± 5% and 70 ± 5%, respectively (Figure 1D,E) whereas PANX2 and PANX3 expressions remained unaffected (Figure S1). Taken together, these results show that PANXs are expressed in HDLECs. Since PANX1 is the prevalent isoform and its expression is specifically modulated by VEGF-C, PANX1 is likely to be involved in lymphatic function.

### 2.2. Pharmacological Inhibitors of Pannexin-1 Modulate In Vitro Lymphangiogenesis

**Figure 2:**
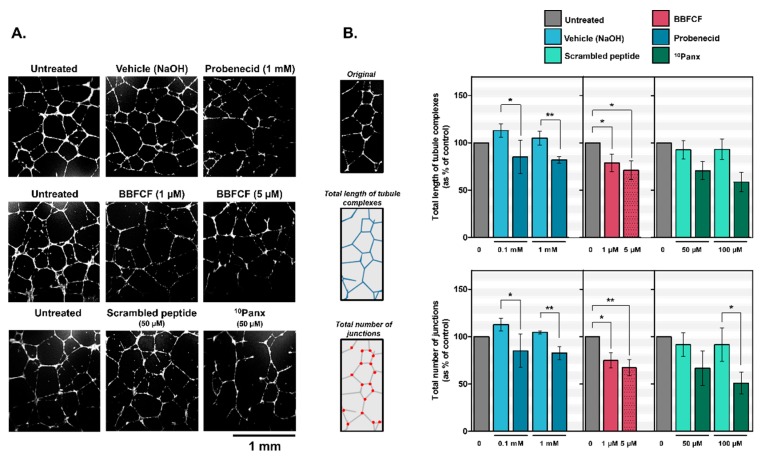
Inhibition of capillary-like formation in HDLECs by pharmacological inhibitors of Pannexin-1. (**A**) Representative images of capillary network formation by HDLECs seeded on Matrigel and treated with Probenecid, Brilliant Blue FCF or mimetic peptide ^10^Panx; (**B**) Quantitative analysis for total length of tubule complexes and for total number of junctions in control and treated HDLECs. Data represent the mean ± SD from three independent experiments conducted in triplicate. * *p* < 0.05 and ** *p* < 0.01.

To investigate whether PANX1 might be involved in lymphangiogenesis we used the in vitro tube-formation assay which is a well-established test based on the ability of LECs to form three-dimensional capillary-like network when seeded on basement membrane extracts (Figure 2A, untreated). Treatment of HDLECs with Probenecid or Brilliant Blue FCF resulted in a disorganized tubular network (Figure 2A). Tube length complexes were significantly inhibited by 28 ± 4% and 20 ± 3% with probenecid at 0.1 and 1 mM respectively and inhibited by 21 ± 3% and 29 ± 4% with Brilliant Blue FCF at 1 and 5 µM respectively (Figure 2B). Number of junctions were also significantly inhibited by 28 ± 4% and 22 ± 3% with probenecid at 0.1 and 1 mM respectively and inhibited by 25 ± 4% and 33 ± 6% with Brilliant Blue FCF at 1 and 5 µM, respectively (Figure 2B).

The implication of PANX1 in this process was confirmed by a second set of experiments using the mimetic inhibitory peptide ^10^Panx instead. Results showed that the lengths of the capillary-like complexes and number of junctions in the HDLECs treated with ^10^Panx at 50 or 100 µM were respectively 22 ± 2% or 34 ± 3% shorter and 25 ± 2% or 40 ± 4% lower than those observed in the control group (Figure 2B). 

### 2.3. Pannexin-1 Silencing Inhibits In Vitro Lymphangiogenesis

**Figure 3:**
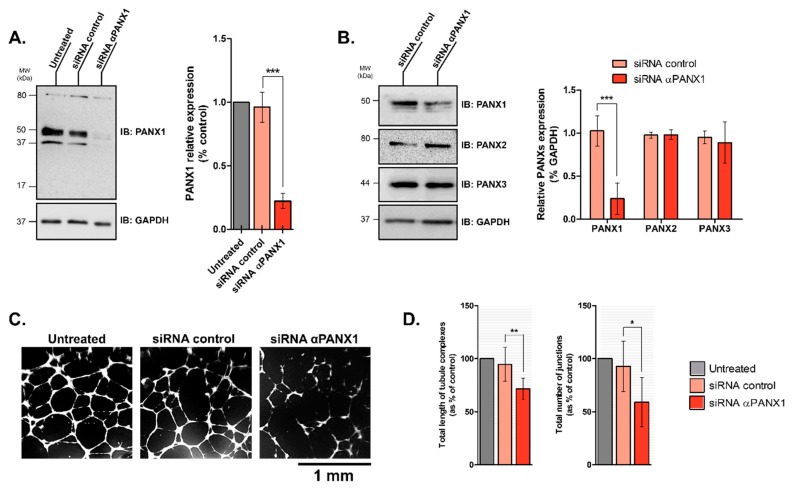
Silencing Pannexin-1 expression affects capillary-like formation by HDLECs. (**A**) Representative immunoblots of HDLECs extracts prepared 48 h after transfection with either the control or the PANX1-specific siRNAs. GAPDH blot served as the loading control; Bar graph shows the quantification of PANX1 expression loss 48 h after siRNA transfection. Data represent the mean ± SD from four independent experiments; (**B**) Representative immunoblots and densitometric quantification of PANX1, PANX2 and PANX3 expression from HDLECs extracts prepared 48 h after transfection with either the control or the PANX1-specific siRNAs. GAPDH blot served as the loading control; (**C**) Representative images of tube structure formation in HDLECs on Matrigel after transfection. Cells transfected with PANX1 siRNAs showed defects in capillary network formation; (**D**) Quantitative analysis for total length of tubule complexes and total number of junctions per field in control and PANX1 siRNA-transfected HDLECs. Data represent the mean ± SD from six independent experiments conducted in duplicate. * *p* < 0.05, ** *p* < 0.01 and *** *p* < 0.001.

To confirm the role of PANX1, we decided to inhibit its expression in HDLECs by siRNA silencing. Figure 3A shows that the siRNA significantly inhibited by 80 ± 11% the expression of PANX1 24 h after transfection compared to scramble. We observed no compensation by PANX2 nor PANX3 expression after PANX1 silencing (Figure 3B). Using the tube formation assay, PANX1 siRNA-transfected HDLECs showed less extensive capillary formation compared to control (Figure 3C). Once again, quantitative analyses showed that the total length of tubule complexes and the number of junctions formed by HDLECs were significantly inhibited by 24 ± 4% and 34 ± 3% respectively when PANX1 was silenced as compared with control (Figure 3D).

### 2.4. Pannexin-1 Silencing Inhibits In Vitro HDLECs Invasion but Not Cell Proliferation

**Figure 4:**
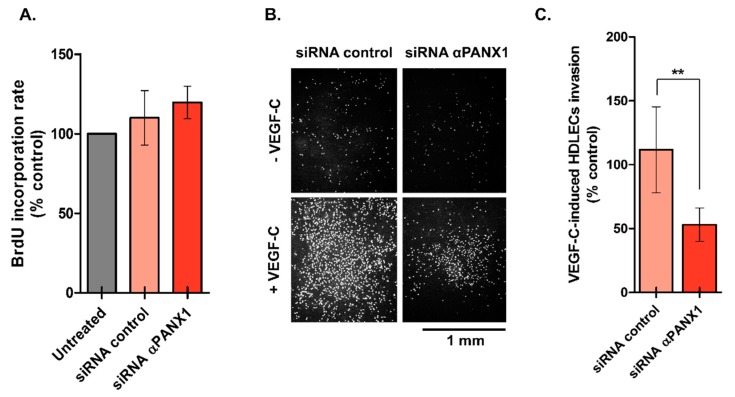
Loss of Pannexin-1 inhibits VEGF-C-mediated invasion of HDLECs. (**A**) HDLECs proliferation measurement 48 h after transfection with either control of PANX1 siRNAs in EGM-V2 media. Data represent the mean from three independent experiments conducted in triplicate (**B**) HDLECs were transfected with either control or PANX1 siRNAs and subjected to Boyden chamber assays in the presence or absence of VEGF-C (100 ng/mL). Representative images of HDLECs that invaded and migrated through the membrane pores after 18 h are shown; (**C**) Bar graph represents the mean number of invading cells. Results are expressed as the mean ± SD of three independent experiments conducted in triplicate. ** *p* < 0.01.

To investigate if PANX1 deficiency in HDLECs affected lymphangiogenesis by modulating cell proliferation, we used the BrdU incorporation assay. The results showed that knockdown of PANX1 had no effect on HDLECs proliferation when LECs were grown in EGM-V2 media (Figure 4A).

Finally, we tested the hypothesis that PANX1 is important for HDLECs invasion and we examined whether invasion of HDLECs induced by VEGF-C is affected by loss of PANX1 using a modified Boyden chamber assay. Figure 4B shows the results of a typical invasion experiment. The quantification revealed a significant inhibition of HDLECs invasion by 59 ± 6% after PANX1 expression silencing compared to scramble (Figure 4C) that can explain the disorganized capillary network we observed previously.

## 3. Discussion

In this present work, we tested the hypothesis that Pannexin-1 is important for human lymphatic endothelial cells to form capillary-like structures for extracellular matrix (ECM)-induced morphogenesis.

We expected PANX1 to be present in human LECs because it is ubiquitously expressed in vivo [2], in several cell lines [47], in human venous ECs [29,30,48] which share a common origin with LECs [36,37] and in murine LECs [45]. Indeed, by quantitative RT-PCR, we found that PANX1 was the most expressed member of the pannexin family in HDLECs. We also observed weak mRNA expression of PANX2 and PANX3 which could confirm their wider expressions in the human body as demonstrated by previous studies [14,15,18,22,23]. Additional work will be required to define the possible role of PANX2 and PANX3 in lymphatic development especially in pathological situations, since no major lymphatic phenotypic abnormalities are observed in adult mice deficient for these PANXs [19,20,32].

Our study reveals that PANX1 exhibited diverse localization patterns in HDLECs. As expected, based on its channel-forming ability, PANX1 localized at the plasma membrane [49,50]. PANX1 has also been found in the perinuclear compartment which has already been observed with endogenous PANX1 in other primary cell cultures or cell lines such as osteoblasts [49] or astrocytes and neurons [51,52,53]. Interestingly, when PANX1 was transfected into human bone marrow ECs, PANX1 localized in the endoplasmic reticulum (ER) and Golgi apparatus [8] and this pattern was observed in vivo in blood ECs of the lens [9]. It is well documented that this pattern reflects the intracellular PANX1 trafficking during which PANX1, as an unglycosylated core (Gly0), is glycosylated in the ER to a high-mannose form (Gly1), and then, in the Golgi to a complex glycosylated form (Gly2), before reaching the plasma membrane [4,46,54]. Our Western blot analysis obtained from HDLECs lysates revealed these multiple species of PANX1 which correlate with PANX1 distribution observed in HDLECs. Another possibility to explain this intracellular localization is that PANX1 may form Ca^2+^-permeable channels in the endoplasmic reticulum as observed in prostate cancer cells [55].

There is then no clear evidence that PANX1 might be essential for proper lymphatic vasculature development. Indeed, neither the Panx1-deficient mice [56,57] nor the only first patient with a PANX1 homozygous germline variant [58] display obvious phenotypes such as lymphedema that would suggest alteration in lymphatic vasculature function. Recently, Molica et al. showed, using double knock-out mice for Panx1 and Apoliprotein E (Apoe) to evaluate Panx1 role in atherosclerosis, that Panx1 in this context is necessary for lymphatic function by contributing to the drainage of interstitial fluid and to the uptake of dietary fat from the gut [47]. Nevertheless, in this study, there was no detail regarding density and morphology of the lymphatic vasculature in this Panx1-/-ApoE-/- mice as for the other Panx1-deficient models. In this present study, using three different strategies to inhibit PANX1, we find that this pannexin is required for in vitro lymphangiogenesis. In addition, we did not observe compensation by PANX2 or PANX3, in PANX1 siRNA-treated HDLECs that failed to form in vitro a well-organized capillaries network. Given the apparent normal lymphatic development in Panx1-deficient mice, it is possible that compensation by Panx2 or/and Panx3 arises in vivo in LECs. This has been observed in muscle [23] and arteries [59] where Panx1 deletion caused an increase in Panx3 expression which can also act as an ATP-release channel [60]. Additional work using Panx1-deficent mice to study the expression of all the members of the pannexin family in the lymphatic vasculature and in isolated LECs will be required to answer this question.

Lymphangiogenesis is a multistep process in which the proliferation and invasion abilities of LECs play a fundamental role. Depending on the cell types, PANX1 may promote [61], decrease [11,62] or have no effect on proliferation [23]. Our work shows that PANX1 knockdown did not change the proliferative rate of HDLECs, which suggests that the inhibition of the in vitro lymphangiogenesis that we observed is not due to an inhibitory effect on the cell cycle. Finally, we focused our work on the role of PANX1 in invasion which is a process that combines both ECM degradation and cell migration. We found out that PANX1, which was up-regulated in HDLECs by VEGF-C, is necessary for the VEGF-C-mediated invasion of HDLECs. Since PANX1 was found at the plasma membrane of HDLECs, it is reasonable to speculate that PANX1 acts as an ATP-release channel to explain its role during this step. We hypothesize that the release of ATP modulates LECs function such as migration through the activation of purinergic receptors as shown for other cell types [63,64]. Once bound to these receptors, ATP might induce Ca^2+^ release from intracellular Ca^2+^ stores, a signaling pathway that has been implicated in LECs migration and lymphangiogenesis [65,66]. LECs are known to express four P2 purinergic receptors (P2RX4, P2RX7, P2RY1 and P2RY11) with high expression of P2RX4 and P2RY1, and interestingly, inhibition of P2RY1 in presence of a specific antagonist impaired ATP-induced migration of HDLECs [67].

Meanwhile, we cannot exclude the hypothesis that PANX1 has channel-independent roles in regulating in vitro lymphangiogenesis. PANX1 directly interacts with the cytoskeleton through its association with actin and Arp2/3 and this association has been shown to modulate cell behavior [50,68,69]. Detailed molecular mechanism of PANX1-driven lymphangiogenesis is still unclear. Future studies using mouse model with lymphatic-specific deletion of PANX1, PANX2 or PANX3 will clarify their relative roles during developmental and pathological lymphangiogenesis.

## 4. Materials and Methods

### 4.1. Antibodies and Reagents

Rabbit polyclonal antibody against PANX1 was purchased from Sigma (HPA016930, St. Louis, MO, USA). Rabbit polyclonal anti-PANX2 was purchased from Santa Cruz Biotechnology (sc-133880, Santa Cruz, CA, USA). Mouse monoclonal anti-PANX3 was from R&D Systems (MAB8169, Minneapolis, MN, USA). Mouse monoclonal anti-GAPDH antibody (5G4) was supplied by HyTest (Turku, Finland). Goat polyclonal HRP-conjugated secondary antibodies anti-Mouse and anti-Rabbit were from Agilent Dako (P044701-2, Santa Clara, CA, USA) and Sigma (A0545), respectively. Goat polyclonal Alexa Fluor 568-conjugated antibodies (anti-Mouse) were purchased from Molecular Probes, Thermo Fisher Scientific (A-11011, Waltham, MA, USA). FITC-Phalloidin (F432) and DAPI (D3571) were from Molecular Probes, Thermo Fisher Scientific. Calcein-AM was purchased from Sigma (C1359). Silencer Pre-designed siRNA against PANX1 (134470) and Silencer Select negative control siRNA (4390843) were obtained from Ambion, Thermo Fisher Scientific. Matrigel**^®^** matrix was obtained from Corning (354234, Corning, NY, USA). Recombinant Human Vascular Endothelial Growth Factor-C (VEGF-C) was obtained from Immunotools (11344692, Friesoythe, Germany) and reconstituted as a 100 µg/mL solution in sterile water. Probenecid (P8761) and Brilliant Blue FCF (80717) were purchased from Sigma and reconstituted as 50 mg/mL solution in 1M NaOH and as 30 mg/mL solution in sterile water, respectively. ^10^Panx mimetic peptide (Trp-Arg-Gln-Ala-Ala-Phe-Val-Asp-Ser-Tyr) and Scrambled ^10^Panx control peptide (Phe-Ser-Val-Tyr-Trp-Ala-Gln-Ala-Asp-Arg) were from Tocris Bioscience (Bristol, UK) and reconstituted as 0.5 mg/mL solution in PBS or water, respectively.

### 4.2. Cell Culture

Primary human dermal lymphatic endothelial cells (HDLECs) were obtained from Promocell (Heidelberg, Germany) and grown in EGM-V2 media which consists of EBM-2 basal media supplemented with 5% FCS and defined supplements such as epidermal growth factor (EGF), basic fibroblast growth factor (bFGF), insulin-like growth factor 1 (long R3 IGF-1), vascular endothelial growth factor A (VEGF-A), ascorbic acid and hydrocortisone. When HDLECs reached 80% confluency, cells were trypsinized following the procedure recommended by Promocell and then plated at 1 × 10^3^ cells/cm^2^. HDLECs were used up to passages 6–8 for all experiments.

### 4.3. Quantitative Real-Time PCR Analysis

1 × 10^5^ HDLECs in 2 mL EGM-V2 medium were seeded in six-well plates and 24 h after, total RNA was extracted using the NucleoSpin RNA XS kit (Macherey-Nagel, Düren, Germany). Reverse transcription was performed with SuperScript II (Invitrogen, Thermo Fisher Scientific, Waltham, MA, USA) from 2 µg of total RNA according to the manufacturer’s instructions. Gene expression was assessed relative to GAPDH by quantitative PCR with the GeneAmp 7000 Sequence Detection System and SYBR Green chemistry (Applied Biosystems, Thermo Fisher Scientific). Human GAPDH, PANX1, PANX2 and PANX3 primer sequences are listed in Table S1. Sensitivity and specificity of each primer couple were checked. For each primers, qPCR was also performed with plasmids containing the cDNA of PANX1, PANX2 and PANX3, serving as positive controls.

### 4.4. Western Blot

1 × 10^5^ HDLECs in 2 mL EGM-V2 medium were seeded in six-well plates. 24 h after, cells were lysed with 30 µL extraction buffer (150 mM NaCl, 10 mM Tris-HCl, pH 8.0, 1 mM EDTA, 1 mM EGTA, 1% Triton X-100, 0.5% NP-40, 100 mM sodium orthovanadate) completed with 1× protease inhibitors cocktail (Roche Applied Science, Penzberg, Germany). Protein concentration was measured using DC protein assay kit (Bio-Rad Laboratories, Hercules, CA, USA). Afterwards 20 µg total proteins samples were mixed with an equal volume of 5X SDS gel-loading buffer (150 mM Tris-HCl, pH 6.8, 5% SDS, 12.5% 2-mercaptoethanol, 25% glycerol and 0.025% bromophenol blue). Proteins were resolved using 10% SDS-PAGE gels and transferred to PVDF membranes (Merck Millipore, Darmstadt, Germany). Membranes were blocked with 5% non-fat powdered milk in Tris-buffered saline-Tween (TBS-T; 25 mM Tris-HCl, pH 8.0, 15 mM NaCl, 0.01% Tween 20) for 3 h and incubated overnight at 4 °C with primary antibodies (anti-PANX1, 1:800; anti-PANX2, 1:350; anti-PANX3, 1:1000). Membranes were then washed with TBS-T and incubated with corresponding secondary horseradish peroxidase-conjugated antibodies (1:10,000) for 1 h. The anti-GAPDH antibodies are used as the loading control (1:10,000). Immunodetection was performed using chemiluminescent substrate Luminata Forte (Merck Millipore) and LAS-3000 imaging system (Fujifilm, Tokyo, Japan). Densitometric analysis of signals was carried out using ImageJ software (version 1.39o, National Institutes of Health, Bethesda, MD, USA). Preliminarily to expression experiments, positive controls samples were used to further validate the banding pattern of each antibody (Figure S2).

### 4.5. Immunofluorescence

HDLECs were plated on 0.1% gelatin-coated sterile glass coverslips in removable 12 well chambers (Ibidi GmbH, Martinsried, Germany) at 2 × 10^4^ cells/well in EGM-V2. After 48 h, HDLECs were washed with 1X PBS and fixed in 4% PFA in 1X PBS at room temperature (RT) for 10 min. HDLECs were then washed with 1X PBS and coverslips were blocked with 1% BSA, 1% Triton X100 in 1X PBS at RT for 1 h. HDLECs were incubated overnight at 4 °C with anti-PANX1 primary antibodies prepared in blocking buffer at 1:100 dilution. A negative control was performed by omitting the primary antibody. The following day, HDLECs were incubated with Alexa Fluor 555 donkey anti-goat secondary antibodies (1:500) in blocking buffer for 2 h at RT. F-actin filaments and cell nuclei were stained with 1 nM FITC-phalloidin (Life Technologies, Thermo Fisher Scientific, Waltham, MA, USA) and 100 nM DAPI (Molecular Probes, Thermo Fisher Scientific) in 1X PBS for 30 min at RT. After extensive washing with 1X PBS, cover slips were mounted with Mowiol fluorescent mounting medium and Images were captured using a confocal microscope (Olympus FV1000, Tokyo, Japan). Control experiment omitting primary antibody was also performed.

### 4.6. siRNA Interference

The siRNA sequence used for small-interfering RNA-mediated inhibition of PANX1 was the following: PANX1 siRNA: 5′-AGGAUCCCUGAUUUGAUGCTG-3′. The siRNA sequence for the non-targeting control siRNA was undisclosed by the manufacturer. Transfections were performed using siPORT reagent according to the manufacturer’s instructions. 2 × 10^5^ HDLECs in 2.3 mL EGM-V2 medium were seeded in six-well plates. For each well, 5 µL siPORT Amine agent were diluted into 100 µL Opti-MEM medium and incubated for 10 min at RT. 7.5 µL and 12.5 µL of PANX1 or scramble siRNA at 10 and 1 µM respectively were diluted into Opti-MEM. Diluted siRNA and diluted siPORT Amine agent were mixed, incubated for 10 min at RT and finally dispensed onto HDLECs. Medium was finally removed 24 h after transfection prior experiments. The efficiency of the siRNA knockdown was determined by Western blot analysis 24 and 48 h post-transfection. XTT assay confirmed non-cytotoxic effects of both siPORT and oligos after 24 h treatment on HDLECs (Figure S3).

### 4.7. Cell Proliferation

BrdU incorporation assay was used to monitor the mitogenic effects of the siRNA silencing of PANX1. siRNA-transfected HDLECs were plated in 96-well plates at a density of 6 × 10^3^ cells/well in 100 µL EGM-V2 and after 24 h bromodeoxyuridine (BrdU) was added into wells at a final concentration of 100 µM and incubated overnight at 37 °C. BrdU incorporation rate was measured using “Cell Proliferation ELISA, BrdU colorimetric kit” (Roche Applied Science, Penzberg, Germany) according to the manufacturer’s instructions. Absorbance was measured at 450 nm with a 96-well microplate reader after adding sulfuric acid to stop colorimetric reaction.

### 4.8. Tube Formation

96-well plates were coated with 50 µL/well of cold Matrigel and allowed to solidify for 1 h at 37 °C. HDLECs were trypsinized and 15 × 10^3^ cells in 200 µL of EGM-V2 medium were loaded on the solidified Matrigel in the presence or absence of PANX1 chemical inhibitors, Probenecid (at 1 or 2 mM final) or BBFCF (at 1 or 5 µM final). In a second set of experiments, HDLECs were mixed and loaded on Matrigel with the specific PANX1 mimetic peptide channel blocker, ^10^Panx or the control peptide at 50 or 100 µM. Finally, PANX1 or control siRNA-transfected HDLECs were used in the same conditions than the native HDLECs in this assay. After 20 h, HDLECs were stained with 25 µM calcein-AM and then fixed with 2% PFA in 1X PBS. The 3-dimensional cell organization was photographed using Olympus MVX10 macroscope (objective 1×/1; 22 °C; medium: PBS; Camera: Hamamatsu ORCA-03G; cellSens Dimension Version 1.4 software Olympus, Tokyo, Japan). Capillary-like structures (length of tubule complexes and number of junctions) were quantified by automatic counting in duplicate using the AngioQuant Version 1.33 software. Previously to these experiments we have determined that the concentrations of inhibitors used in this assay on the HDLECs were not cytotoxic (Figure S3).

### 4.9. Endothelial Cells Invasion

HDLECs migration was evaluated using 24-well cell culture inserts with 8-µm pores (BD Biosciences, Franklin Lakes, NJ, USA). 24 h after siRNA transfection, HDLECs were rinsed with EBM-2 media, detached with trypsin and seeded at 5 × 10^4^ cells/100 µL 0.5% FCS EBM-2 onto Matrigel-coated (25 µL of 1 mg/mL) inserts. The inserts were then placed in the 24-well plates containing 500 µL of EBM-2 medium with or without 100 ng/mL recombinant human VEGF-C. The filters were removed following incubation for 17 h at 37 °C and 5% CO_2_ and the Matrigel was wiped with a cotton swab. The invasive HDLECs were fixed in 4% PFA for 10 min prior to staining with DAPI. Filters were mounted with Mowiol fluorescent mounting medium and cells were photographed using an Olympus MVX10 macroscope (objective 2X, Tokyo, Japan). Cells were counted using ImageJ software after binarizing images.

### 4.10. Statistical Analysis

Statistical analyses were carried out on GraphPad Prism 5 software. All reported data are expressed as mean ± SD. ANOVA followed by Bonferroni post-tests was performed for analysis of 3 or more groups. Unpaired Student’s *t*-test (Mann-Whitney) was used when only 2 experimental groups were analyzed.

## 5. Conclusions

In this study, we provide evidences that Pannexin-1 is expressed in human lymphatic endothelial cells and is involved in in vitro lymphangiogenesis.

## Supplementary Materials

Supplementary materials can be found at http://www.mdpi.com/1422-0067/19/6/1558/s1.

## Abbreviations

BrdU	Bromodeoxyuridine
CX	Connexin
DAPI	4′,6-diamidino-2-phenylindole
EBM	Endothelial cell basal medium
ECs	Endothelial cells
EGM	Endothelial cell growth medium
FCS	Fetal calf serum
GAPDH	Glyceraldehyde 3-phosphate dehydrogenase
HDLECs	Human dermal lymphatic endothelial cells
HUVECs	Human umbilical vein endothelial cells
PANX	Pannexin
PBS	Phosphate-buffered saline
PFA	Paraformaldehyde
RT	Room temperature
RT-PCR	Reverse transcription-polymerase chain reaction
siRNA	Small-interfering RNA
SMCs	Smooth muscle cells
TBS	Tris-buffered saline
VEGF-C	Vascular endothelial growth factor-C
