# Supplementary material for: Pannexin-1 in Human Lymphatic Endothelial Cells Regulates Lymphangiogenesis
Source: Int J Mol Sci. 2018 May 24;19(6):1558. doi: 10.3390/ijms19061558 (PMC6032340; doi:10.3390/ijms19061558)
Supplement: Supplementary file 1 [file ijms-19-01558-s001.pdf]

Supplementary

Pannexin-1 in Human Lymphatic Endothelial Cells Regulates Lymphangiogenesis

Jonathan Boucher, Claire Simonneau, Golthlay Denet, Jonathan Clarhaut, Annie-Claire Balandre, Marc Mesnil, Laurent Cronier and Arnaud Monvoisin

Table S1: qRT-PCR primers

Gene ID	Forward	Reverse
PANX1	5'-CGTGACCTTGACATGAGAGATG-3'	5'-CTGCTCCACAATTGGGTACTT-3'
PANX2	5'-TTCTGCGACATCAACATCCT-3'	5'-ACCACGTTGTCGTACATGAG-3'
PANX3	5'-AAGGCTCGGAAAGAACGATAC-3'	5'-GGAGGTGAAGATGAGCAAGAG-3'
GAPDH	5'-TGCACCACCAACTGCTTAGC-3'	5'-GGCATGGACTGTGGTCATGAG-3'

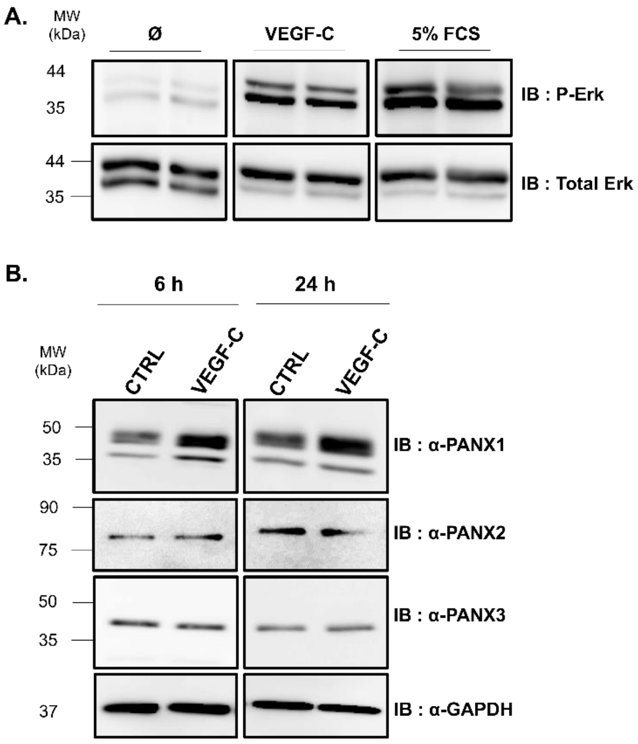

Figure S1. VEGF-C modulates PAN1 expression without interfering with PANX2 and PANX3 expressions. **(A)** We validated VEGF-C efficiency by showing increased ERK activation after stimulation of serum-starved HDLECs with VEGF-C (100 ng/mL). FCS was used as positive control. The blots are representative of two independent experiments. **(B)** PANX1 expression was increased after 6 and 24 h of incubation with VEGF-C (100 ng/mL). VEGF-C did not seem to regulate PANX2 and PANX3 expressions. The blots are representative of two or three independent experiments ($N = 3$ for PANX1, $N = 2$ for PANX2 and PANX3).

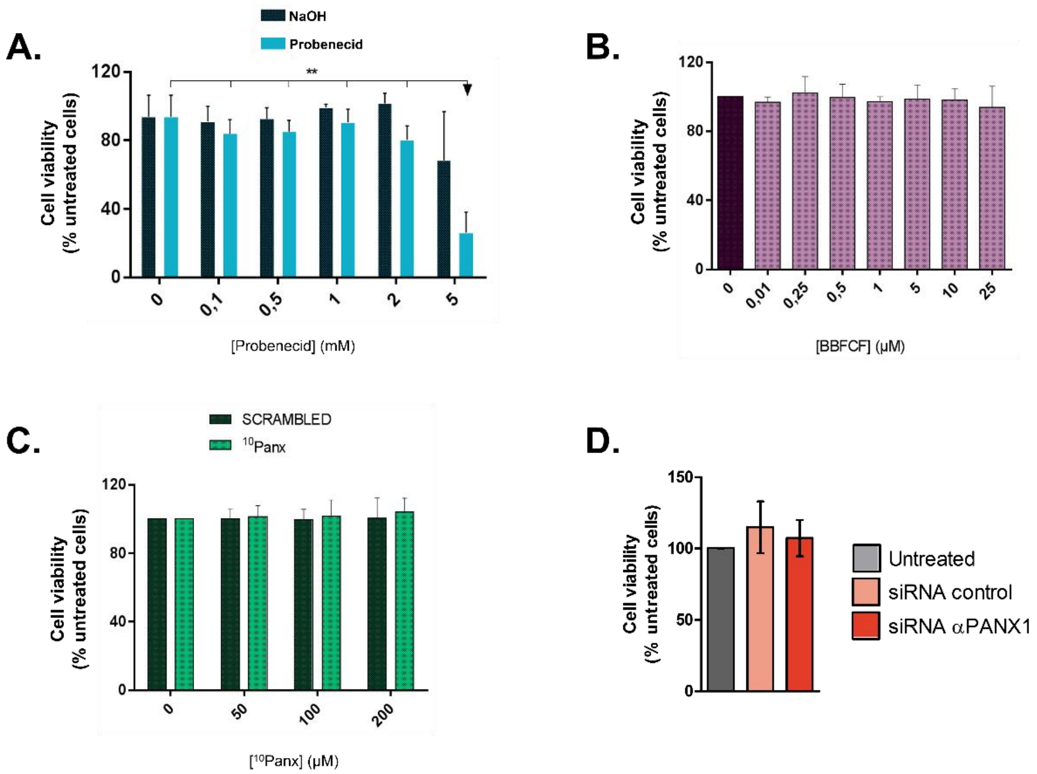

Figure S2. HDLECs viability assay at different concentrations of Probenecid, BBFCF and [10]Panx. HDLECs viability was assessed in 96-well plates with the Cell Proliferation Kit II (XTT, Sigma-Aldrich) after 24 h incubation with increasing concentrations of the different inhibitors or after siRNA treatments. **(A)** Probenecid had no cytotoxic effects up to the dose of 2 mM. **(B)** BBFCF and **(C)** [10]Panx had no cytotoxic effects even at the maximum tested concentrations of 25 µM and 200 mM respectively. (**D**) siRNA control and anti-PANX1 transfections had no cytotoxic effects compared to untreated cells. Data obtained from four independent experiments performed in quadruplicate. ** $p < 0.01$.

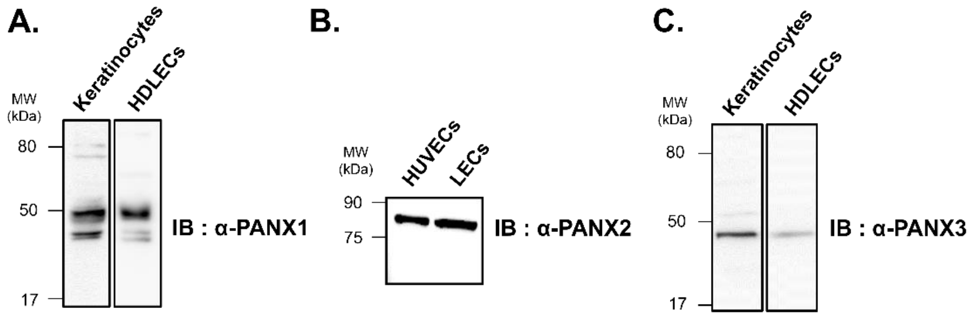

Figure S3. Determination of the sensitivity of antibodies to PANXs. Determination of the sensitivity of antibodies to PANXs. Lysates from human primary keratinocytes and Human Umbilical Vein Endothelial Cells (HUVECs) were used as a positive control to assess the sensitivity of PANXs antibodies directed against (**A**) PANX1, (**B**) PANX2 or (**C**) PANX3.
